# Antimicrobial and Antivirulence Action of *Eugenia brejoensis* Essential Oil *in vitro* and *in vivo* Invertebrate Models

**DOI:** 10.3389/fmicb.2020.00424

**Published:** 2020-03-19

**Authors:** Clovis Macêdo Bezerra Filho, Luís Cláudio Nascimento da Silva, Márcia Vanusa da Silva, Anders Løbner-Olesen, Carsten Struve, Karen Angeliki Krogfelt, Maria Tereza dos Santos Correia, Maria Luiza Vilela Oliva

**Affiliations:** ^1^Biochemistry Department, Federal University of Pernambuco, Recife, Brazil; ^2^Biochemistry Department, Federal University of São Paulo, São Paulo, Brazil; ^3^Programa de Pós-graduação em Biologia Microbiana, CEUMA University, São Luís, Brazil; ^4^Department of Biology, University of Copenhagen, Copenhagen, Denmark; ^5^Department of Bacteria, Parasites and Fungi, Staten Serum Institut, Copenhagen, Denmark; ^6^Department of Science and Environment, Roskilde University, Roskilde, Denmark

**Keywords:** *Caenorhabditis elegans*, *Galleria melonella*, infections models, multidrug resistance, natural products, *Staphylococcus aureus*, virulence factors

## Abstract

*Eugenia brejoensis* L. (Myrtaceae) is an endemic plant from caatinga ecosystem (brazilian semi-arid) which have an *E. brejoensis* essential oil (EbEO) with reported antimicrobial activity. In this work, *in vitro and in vivo* models were used to characterize the inhibitory effects of EbEO in relation to *Staphylococcus aureus.* EbEO inhibited the growth of all tested *S. aureus* strains (including multidrug resistance isolates) with values ranging from 8 to 516 μg/mL. EbEO also synergistically increased the action of ampicillim, chloramphenicol, and kanamycin. The treatment with subinhibitory concentrations (Sub-MIC) of EbEO decreased *S. aureus* hemolytic activity and its ability to survive in human blood. EbEO strongly reduced the levels of staphyloxanthin (STX), an effect related to increased susceptibility of *S. aureus* to hydrogen peroxide. The efficacy of EbEO against *S. aureus* was further demonstrated using *Caenorhabditis elegans* and *Galleria mellonella*. EbEO increased the lifespan of both organisms infected by *S. aureus*, reducing the bacterial load. In addition, EbEO reduced the severity of *S. aureus* infection in *G. mellonella*, as shown by lower levels of melanin production in those larvae. In summary, our data suggest that EbEO is a potential source of lead molecules for development of new therapeutic alternatives against *S. aureus*.

## Introduction

*Staphylococcus aureus* is the etiologic agent of a wide spectrum of clinical conditions ranging from superficial skin infections and soft tissue infections to severe sepsis (Asgeirsson et al., 2018; Mcneil and Fritz, 2019; Turner et al., 2019). The indiscriminate use of antibiotic has induced the emergence of multidrug resistant (MDR) strains that have spread from hospital settings to different environmental and victimized various individuals (Mcneil and Fritz, 2019; Turner et al., 2019).

Indeed, both hospital- and community-associated *S. aureus* strains display complex combinations of virulence and resistance genes and have been related to increased ratios of morbidly and mortality and represent serious concerns for global systems worldwide (De Carvalho et al., 2019; Liang et al., 2019; Vestergaard et al., 2019). The major drugs currently used against methicillin-resistant *S. aureus* (MRSA) are vancomycin, teicoplanin, linezolid, and daptomycin (Werth et al., 2014); however, isolates with resistance or low susceptibility have been detected for all these drugs (Endimiani et al., 2011; Kos et al., 2012; Capone et al., 2016; Bakthavatchalam et al., 2017). Taken together, these data justify the search for new alternatives for the treatment of infections caused by *S. aureus*, and plant-derived products are highlighted as interesting candidates (Dos Santos et al., 2016; Mittal et al., 2018).

Plants are known to produce a large variety of molecules with interest for pharmaceutic and food industries, among them are the volatile compounds present in the essential oils (EOs) (Alves et al., 2017; Mittal et al., 2018; Stevanovic et al., 2018; Taghavi et al., 2018; Rao et al., 2019). EOs are complex mixture of secondary metabolites produced by aromatic plants usually involved in their protection against pathogens (Mittal et al., 2018). Several EOs have been demonstrated as efficient antimicrobial agents able to inhibit different MDR strains and to improve the activity of antibiotics (Langeveld et al., 2014; Mittal et al., 2018; Rao et al., 2019). The EOs components can interact with multiple targets in bacteria such as membrane, proteins synthesis, as well as inhibit efflux pumps and virulence related pathways (such as biofilm formation and toxin production) (Dos Santos et al., 2016; Kim et al., 2016; Kong et al., 2016b; Kang et al., 2018; Mittal et al., 2018; Rubini et al., 2018; Espinoza et al., 2019).

*Eugenia brejoensis* is an EO-bearing plant from *Mytaceae* family which has been described as an endemic species in Brazil (present in the states of Pernambuco, Sergipe, Alagoas, Paraíba, Espirito Santo) (Mazine and Souza, 2008; Giaretta and Peixoto, 2014; Mendes et al., 2018). The *E. brejoensis* EO (EbEO) is mainly composed by sesquiterpenes (such as δ-cadinene, β-caryophyllene, and α-muurolol) (Da Silva et al., 2015). EbEO has shown inhibitory actions against *Aedes aegypti* larvae (Da Silva et al., 2015) and *Trypanosoma cruzi* (Oliveira De Souza et al., 2017). In relation to antibacterial activity, nanoemulsions of EbEO were able to inhibit the growth of *Pseudomonas fluorescens* (Mendes et al., 2018). The present work aims to demonstrate the efficacy of EbEO against *S. aureus* using *in vitro* and *in vivo* invertebrate models (*Caenorhabditis elegans* and *Galleria mellonella*).

## Materials and Methods

### Plant Material

Leaves of *E. brejoensis* were collected at *Parque Nacional do Catimbau* (Pernambuco, Brazil) on dry season (September, 2015). All the plant material was processed following the usual techniques in taxonomy and deposited in the Herbarium of *Instituto Agronomico de Pernambuco* (voucher access number: IPA 84.033). The EO was obtained from leaves of *E. brejoensis* (EbEO) by hydrodistillation as previously reported (Da Silva et al., 2015). The oil used in this study was characterized by gas chromatography–mass spectrometry (GC/MS) and the chemical profile was published by Da Silva et al. (2015).

### Bacterial Strains Used in the Study

The standard strain *S. aureus* ATCC 29312 was used in most of the assays. The antimicrobial activity of EbEO was further analyzed against a collection of clinical isolates of *S. aureus* deposited in the Microbial Collection of *Departamento de Antibioticos* from *Universidade Federal de Pernambuco* (UFPEDA). The antibiotic resistance profile of each strain is shown in Table 1. The strains used in the present study are part of Dr. Anders Løbner-Olesen collection at University of Copenhagen. The expression of virulence- and SOS-related genes (*hla*, *spa*, or *recA*) was performed using strains carrying the targeted genes fused with *lacZ* (which encodes for β-galactosidase) (Nielsen et al., 2010; Gottschalk et al., 2013). The strains in Table 1 were kindly shared by Prof. Hanne Ingmer, Copenhagen University.

**TABLE 1:** Antimicrobial effects of EbEO in association to antibiotics.

***S. aureus***	**Source**	**Resistance profile**	**MIC (μg/mL)**
ATCC 29213	Standard strain	–	128
UFPEDA 02 (= ATCC 6538)	Standard strain	–	256
UFPEDA 659	Catheter tip	NAL/OXA	128
UFPEDA 671	Bone fragment	AMI/AMP/CIP/CLI/CLO/GEN/NAL/OXA/TET/TRI	512
UFPEDA 679	Surgical wound secretion	AMI/CLI/AMP/CFL/CFZ/NAL/OXA/VAN	128
UFPEDA 683	Purulent exudate	AMI/AMP/CIP/CFL/CFO/CFZ/CLI/CLO/CPM/CRX/CTX/GEN/NAL/OXA/TRI/VAN	8
UFPEDA 691	Catheter tip	CIP/CLO/NAL	128
UFPEDA 705	Surgical wound	AMP/CFL/CFO/CPM/CRX/NAL/NIT/OXA/GEN	256
UFPEDA 726	Nasal secretion	AMP/CIP/CLO/GEN/OXA/TRI	128
UFPEDA 731	Surgical wound secretion	AMP/CFL/CFO/CLI/CLO/CRX/CIP/GEN/NAL/OXA/TRI	512
UFPEDA 802	Nasal secretion	AMI/AMP/OXA/CFL/CFO/CFZ/CIP/CLI/CLO/CPM/CRX/CTX/GEN/NAL/TET/TRI	512

*AMI: amikacin; AMP: ampicillin; CFL: cephalothin; CFZ: cefazolin; CPM: cefepime; CFO: cefoxitin; CIP: ciprofloxacin; CLI: clindamycin; CLO: chloramphenicol; CRX: cefuroxime; CTX: cefotaxime; GEN: gentamicina; IMI: imipenem; MER: meropenem; NAL: nalidixic acid; NIT: nitrofurantoin; OXA: oxacillin; TET: tetracycline; TRI: trimethoprim; VAN: vancomycin. MICs were obtained using the microdilution method as described.*

### Determination of Minimum Inhibitory Concentration

The antimicrobial activity of EbEO was determined against the standard strains *S. aureus* ATCC 29312 and clinical isolates (Table 1). Serial dilutions (1024 to 2 μg/mL) of EbEO were prepared in 96-wells plates containing Luria–Bertani (LB) broth. Each well received 10 μL of a microbial suspension [bacterial load of approximately 1.0 × 10^7^ colony forming units per milliliter (CFU/mL) for each well]. The plates were incubated at 37°C, and after 24 h each well received 30 μL of 0.03% resazurin sodium solution (Sigma–Aldrich^®^). Following, the plates were incubated for 40 min and the minimum inhibitory concentration (MIC) was defined as the lowest concentration capable of inhibiting bacterial growth (as evaluated color change).

### Time-Kill Studies

Overnight cultures of *S. aureus* ATCC 29312 were diluted 1:100 in LB broth and placed in a shaking water bath at 37°C until an optical density at 600 nm (OD_600_) of 0.1 was reached. This microbial suspension was distributed in fresh LB broth containing EbEO (128 or 1024 μg/mL; corresponding to 2 × MIC or 8 × MIC, higher concentrations was applied to verify action of concentrations greater than MIC50). Bacteria treated with ciprofloxacin (2 μg/mL; 2 × MIC) or without treatment were used as positive and negative controls, respectively. Cell growth was monitored in specific time points (0, 1, 2, 3, 4, and 5 h) by plating 4 μL of 10-fold-diluted suspensions from each tube in quadruplicate. The plates were incubated at 37°C for 24 h. After this period, the colonies were counted for the calculation of CFU/mL.

### Combinatory Effects

The interaction between EbEO and some important clinical used drugs (ampicillin—25 μg/mL, ciprofloxacin—0.78 μg/mL, chloramphenicol—12.5 μg/mL, erythromycin—0.39 μg/mL, and kanamycin—6.25 μg/mL) were evaluated using checkerboard assay against *S. aureus* ATCC 29312. Fractional inhibitory concentration index (FICI) was assessed algebraically using the following equation:

FICI=FIC+EbEOFIC=D(EbEO/MIC)EbEO+(D/MIC)D

Where “EbEO” is the concentration (μg/mL) of EbEO in a given well, and MIC_EbEO_ represents the control MIC of EbEO alone. “D” is the concentration of the tested drug in a given well, and MIC_D_ represents the MIC of the tested drug alone. The interactions were defined as: (i) synergistic if FICI ≤ 0.5; (ii) additive if 0.5 < FICI ≤ 1; (iii) non-interaction if 1 < FICI < 4; and (iv) antagonistic if FICI ≥ 4 (Da Silva et al., 2016).

### Expression of Virulence- and SOS-Related Genes Through β-Galactosidase Activity

The effects of EbEO on the expression of genes related to virulence and SOS pathways were performed using *S. aureus* 8325-4 derivative strains carrying the targeted genes (*hla*, *spa*, or *recA*) fused with *lacZ* (which encodes for β-galactosidase) (Vestergaard et al., 2015). In all assays, each strain was exponentially grown in LB medium until an OD_600_ between 0.1 and 0.2. Cells were treated with EbEO (64 μg/mL, 0.5 × MIC), and permeabilizated by toluene (1 mL) after 3 h. The β-galactosidase activity was measured using ONPG (*ortho*-nitrophenyl-β-galactoside; Sigma–Aldrich). In the assays for *recA* expression, ciprofloxacin (0.5 μg/mL) was used as positive control.

### Evaluation of Staphyloxanthin (STX) Inhibition

Overnight cultures of *S. aureus* ATCC 29312 were diluted (1:100) in LB medium and samples (1 mL) of this suspension were incubated with sub-inhibitory concentrations of EbEO (64, 32, and 16 μg/mL, corresponding, respectively, to 0.125 × MIC, 0.25 × MIC, and 0.5 × MIC). After overnight incubation at 37°C, the tubes were centrifuged (10,000 r/min for 10 min), suspended with 1 mL of phosphate-buffered saline (PBS), and re-centrifuged. Bacteria pellets were then photographed. Following the total carotenoid pigments [including staphyloxanthin (STX)] in each pellet were extracted using in methanol (0.2 mL) and incubated for 3 min at 55°C. The methanol phase (supernatant) and cell debris were separated by centrifugation (10,000 r/min for 10 min) and the pellets were submitted to entire pigment extraction procedure three more times. Finally, the absorbance was determined at 465 nm (Silva et al., 2017).

### Effect of EbEO on Susceptibility of *S. aureus* to Hydrogen Peroxide

Microbial suspension (standardized at OD_600_ = 1.0; Liu et al., 2005) prepared from overnight cultures of *S. aureus* ATCC 29312 were incubated with sub-inhibitory concentrations of EbEO (64, 32, and 16 μg/mL, corresponding, respectively, to 0.125 × MIC, 0.25 × MIC, and 0.5 × MIC). Each culture received H_2_O_2_ to reach a final concentration of 1.5% (v/v) for 60 min and were incubated at 37°C. The percentage of cells surviving the stresses was calculated as CFU/mL remaining after each stress divided by the initial CFU/mL.

### Anti-hemolytic Evaluation

*Staphylococcus aureus* ATCC 29312 overnight cultures were diluted at 1:100 in fresh LB and cultured with or without sub-inhibitory concentrations of EbEO (64 and 32 μg/mL) at 37°C. After 16 h (to guarantee a constant number of bacteria present in experiment), 50 μL of culture supernatant was added to 1 mL of 3% human erythrocytes solution. The mixture was incubated at 37°C for 1 h with 250 r/min shaking. The supernatant was collected by centrifugation at 10,000 r/min for 10 min, and the optical density was measured at 540 nm.

### Bacterial Activity in Whole Blood

To verify whether EbEO could enhance the antimicrobial action in whole blood, the *S. aureus* ATCC 29312 (diluted in LB medium; 1:100) was grown in the presence of EbEO (64 and 32 μg/mL) at 37°C with shaking of 250 r/min. After 6 h, the *S. aureus* suspension (62.5 μL) with EbEO (64 or 32 μg/mL) were mixed with freshly drawn human whole blood (0.5 mL) and re-incubated in the conditions described above for 2 h. The samples were plated in LB agar and the survival was measured by counting viable colonies (expressed as CFU/mL) (Lee et al., 2013).

### Infectious Model With *Caenorhabditis elegans*

The infection model was performance using *C. elegans* AU37, a temperature-sensitive sterile strain [sek-1(km4); glp-4(bn2) I; MAPK kinase deficiency] (Jakobsen et al., 2013). The strain was propagated on nematode growth medium (NGM) containing *Escherichia coli* OP50 as food source. Prior each assay, the worms were age-synchronized by bleaching with alkaline hypochlorite and sodium hydroxide. The released embryos were placed on NGM plates at 25°C (this temperature does not allow the reproduction) until reached the young adult stage. At this time, 15 larvae were transferred to 24-wells plates containing the M9 liquid medium and overnight culture *S. aureus* ATCC 29312 (grown in LB medium containing 10 μg/mL cholesterol) in a ratio of 4:1 (v/v) (Kong et al., 2014). EbEO (128, 64, and 32 μg/mL) was added and the worm longevity was assessed every day. OP 50 was used with positive control (worm and *E. coli*) and *S. aureus* was used with negative control (worm and ATCC 29312). The animals were classified as dead when they did not present spontaneous movement or response after stimulation with a platinum loop. All experiments were performed according to Wormbook (Stiernagle, 2006).

### Infectious Model With *Galleria mellonella*

*Galleria mellonella* larvae (∼200 mg) were randomly distributed in three experimental groups (*n* = 10) with or without oil treatment. Two groups were infected by injection of 10 μL of a recent *S. aureus* ATCC 29312 suspension (1.0 × 10^5^ CFU; different bacterial concentrations was previously optimized to guarantee an inicial sublethal load to this assay), into the last left proleg, followed by incubation at 37°C. After 2 h, one of these groups received 10 μL of 128 μg/mL EbEO (resulting in dose of 6.4 mg/kg). Larvae treated with PBS (vehicle) were used as positive control.

### Quantification of *S. aureus* Load in *G. mellonella* Hemolymph

*Galleria mellonella* larvae were infected with *S. aureus* ATCC 29312 and treated with EbEO as described above. Each day, a total of five larvae were cut in the cephalocaudal direction with a scalpel blade and squeezed to remove the hemolymph. Each sample was 10-fold-diluted in PBS and 4 μL was plated on LB agar. After 24h-incubation at 37°C, the colonies were enumerated, and the results expressed as CFU/mL.

### Quantification of Hemolymph Melanization

Melanization test concerns the production of this pigment under stress conditions. *G. mellonella* (*n* = 10) were infected with *S. aureus* ATCC 29312 (10^6^ cells/larva) aimed to evaluate melanization as a response to higher concentrations of bacteria (stress) and to quantify this response as a function of the melanin/time correlation after being treated with 6.4 mg/kg EbEO. After the incubation (1 or 3 h), the hemolymph was collected and diluted (1:10; v/v) in cold PBS. The cells suspensions were centrifuged at 12,000 r/min and the absorbance of each supernatant was determined at 465 nm.

### Statistical Analysis

All assays were performed in triplicate in at least two independent experiments. Statistical analyses were performed using the software GraphPad Prism version 7.^1^ Data were analyzed by one-way, two-way analysis of variance (ANOVA), and Tukey test. A *p*-value of < 0.05 was considered as statistically significant. Differences in the survival were determined using the Kaplan–Meier method and log-rank test was used to compare survival curves.

## Results

### EbEO Enhances the Activity of Drugs Toward *S. aureus*

Initially, we performed a microdilution-based assay to evaluate antimicrobial activity of EbEO against all tested *S. aureus* strains, including those with multidrug resistance profile (MDR strains). This oil inhibited the standard *S. aureus* ATCC 29312 with an MIC of 128 μg/mL, while the MIC values for the other strains ranged from 8 to 516 μg/mL. The MIC_50_ (concentration able to inhibit 50% of the tested strains) was 128 μg/mL (Table 1).

**FIGURE 1:**
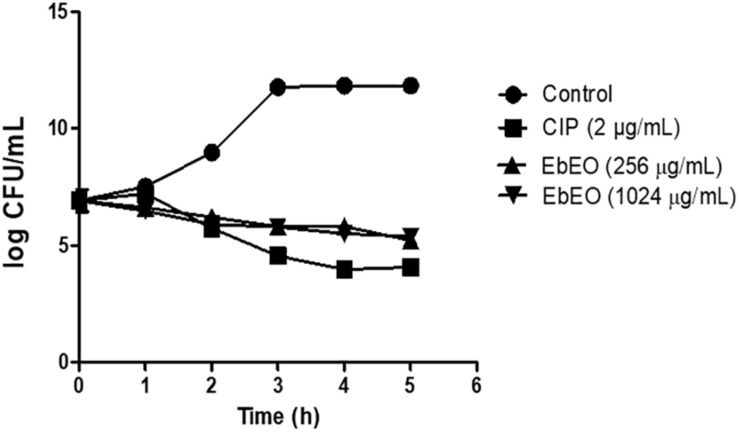
Effects of *Eugenia brejoensis* essential oil (EbEO) on growth of *Staphylococcus aureus*. *S. aureus* ATCC 29312 was treated with 2 × MIC (256 μg/mL) or 8 × MIC EbEO (1024 μg/mL). Bacteria treated with ciprofloxacin (CIP at 2 μg/mL; 2 × MIC) or without treatment were used as positive and negative controls, respectively. Samples were taken every hour to assess bacterial viability.

**FIGURE 2:**
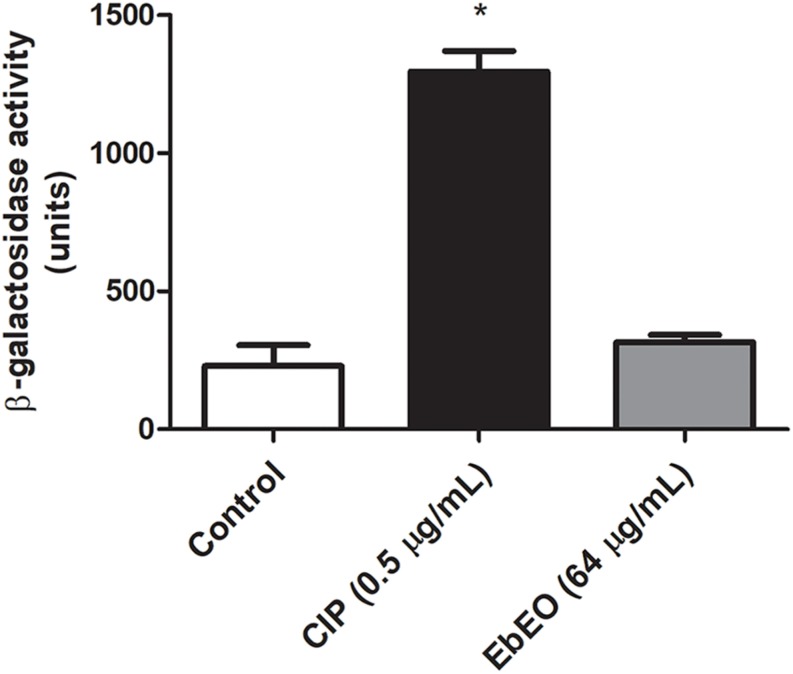
Effects of *Eugenia brejoensis* essential oil (EbEO) on expression of *recA*. The expression of *recA* was performed using a derivative *S. aureus* 8325-4 strain carrying a *recA:lacZ* fusion. Bacteria were treated with EbEO (64 μg/mL; 0.5 × MIC) or ciprofloxacin (CIP at 0.5 μg/mL; 0.5 × MIC) for 3 h. β-Galactosidase activity was measured using ONPG. * Indicates significant differences in relation to control cells (*p* < 0.05).

Following, a time-kill study was performed using EbEO at 128 μg/mL (2 × MIC) or 1024 μg/mL (8 × MIC). Both oil concentrations were able to inhibit the growth of *S. aureus* ATCC 29312 without any significant differences among them. After 3 h, the oil induced reductions of 50% in the number of viable colonies when compared to untreated cells. The oil exhibited a profile, similar to the observed from ciprofloxacin (0.5 μg/mL) (Figure 1). Further, the action of EbEO was not associated with an increased expression of *recA*, the first gene related with the activation of SOS response, a pathway involved in the DNA repair (Vestergaard et al., 2015) (Figure 2).

Next, we determined whether EbEO could improve the action of some antibiotics (from different classes) toward *S. aureus*. EBEO synergistically increased the action of ampicillin (ΣFIC: 0.45), chloramphenicol (ΣFIC: 0.15), and kanamycin (ΣFIC: 0.075), while it had additive effects with ciprofloxacin (ΣFIC: 0.6) and erythromycin (ΣFIC: 0.6).

### EbEO Affects the Expression of *Quorum Sensing*-Related Genes of *S. aureus* and Its Hemolytic Activity

**FIGURE 3:**
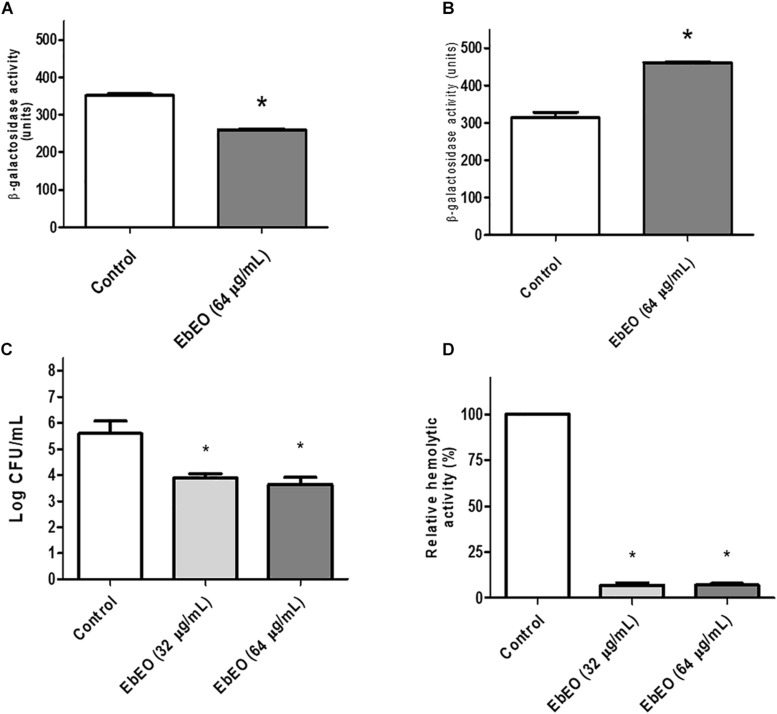
Influence of *Eugenia brejoensis* essential oil (EbEO) on some virulence properties of *S. aureus.* **(A)** Effects of EbEO on expression of *hla* using *S. aureus* 8325-4 derivative strains carrying *hla:lacZ*. **(B)** Effects of EbEO on expression of *spa* using *S. aureus* 8325-4 derivative strains carrying *spa:lacZ*. **(C)** Interference of EbEO on hemolytic activity of *S. aureus*. **(D)** Inhibition of EbEO on *S. aureus* survival on whole blood. * Indicates significant differences in relation to control cells (*p* < 0.05).

Following, we evaluated the effects of EbEO on the expression of two gene of this system (*hla* and *spa* that encode alpha-hemolysin and protein A, respectively). It is expected that a *quorum sensing* inhibitor (QSI) reduces the expression of *hla* and increases the transcriptional levels of *spa* (Nielsen et al., 2010). Based on this, the results indicated the EbEO (at 0.5 × MIC) could alter the expression of *hla* (downregulation) and *spa* (upregulation), suggesting that this oil is QSI (Figures 3A,B).

We further analyzed whether EbEO could decrease the hemolytic activity of *S. aureus* and its ability to survive in human blood. The treatment with EbEO (0.5 × MIC or 0.25 × MIC) strongly inhibited the hemolysis-mediated by *S. aureus* (around 90% of inhibition) (Figure 3C). Similarly, the survival of this pathogen on blood was impaired by subinhibitory concentrations (Sub-MIC) values of EbEO (Figure 3D).

### EbEO Inhibits the Production of Staphyloxanthin and Increases the Susceptibility of *S. aureus* to Hydrogen Peroxide

**FIGURE 4:**
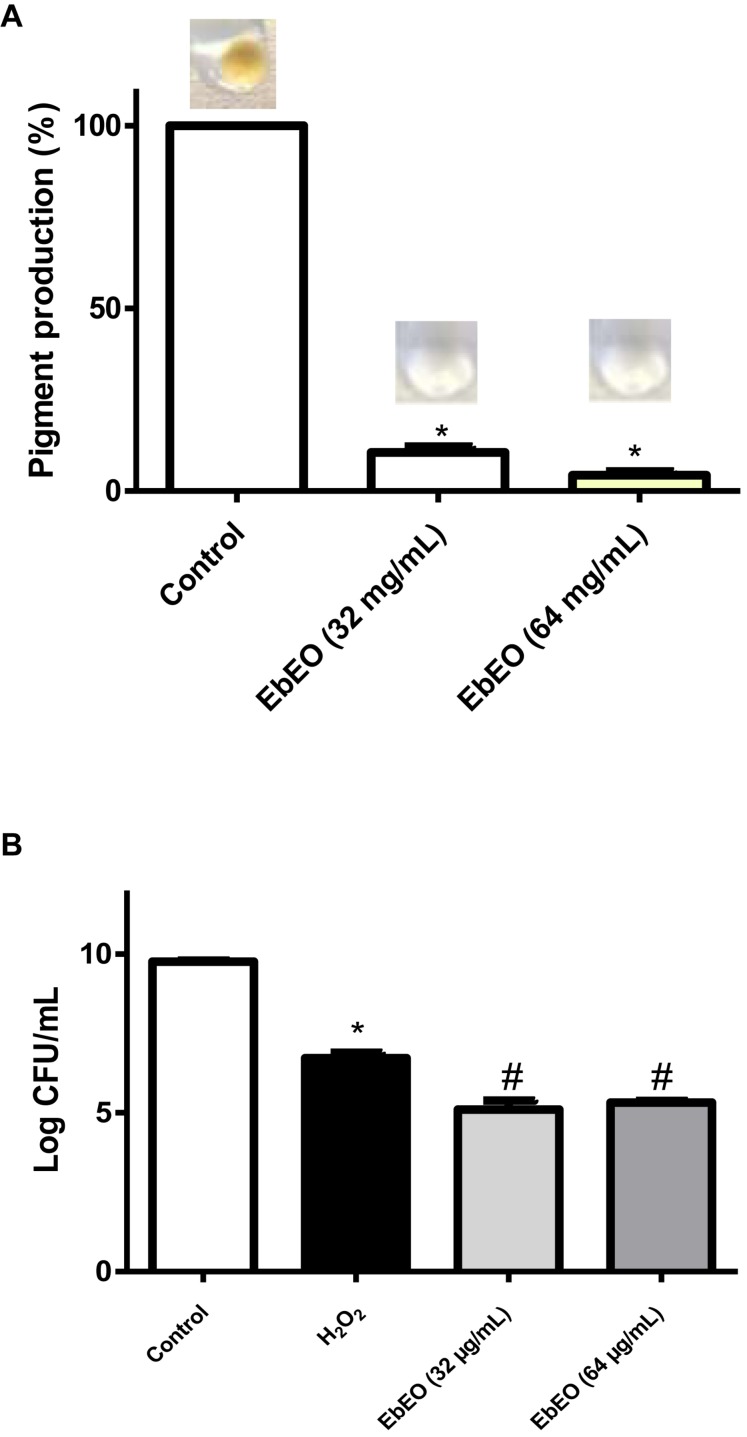
*Eugenia brejoensis* essential oil (EbEO) reduces the staphyloxanthin production and *S. aureus* resistance to hydrogen peroxide. **(A)** EbEO inhibits the staphyloxanthin production. **(B)** The survival of *S. aureus* after oxidative stress is reduced by EbEO. * Indicates significant differences in relation to control cells (*p* < 0.05). # Indicates significant differences in relation to H_2_O_2_ cells (*p* < 0.05).

Figure 4A shows that EbEO strongly reduced the levels of staphyloxanthin in a dose-dependent manner (95.63 ± 0.98 and 89.38 ± 1.49% for EbEO at 64 or 32 μg/mL, respectively). In addition, the treatment with EbEO (at 64 or 32 μg/mL; 0.5 × MIC or 0.25 × MIC) potentialized the toxicity of hydrogen peroxide (1.5% v/v) toward *S. aureus* (Figure 4B). Similar levels of viability reduction were observed for both concentrations (24.12 ± 4.19 and 20.87 ± 0.87% for EbEO at 64 and 32 μg/mL, respectively), when compared to cells treated with hydrogen peroxide alone (*p* < 0.05). These results suggest that the inhibition of staphyloxanthin production by EbEO impaired the antioxidant system of *S. aureus* and increased its susceptibility to oxidant attack. Based on these findings, we decided to evaluate the anti-infective efficacy of EbEO using two alternative experimental models: *C. elegans* and *G. mellonella*.

### EbEO Increases the Lifespan of *C. elegans* Infected by *S. aureus*

**FIGURE 5:**
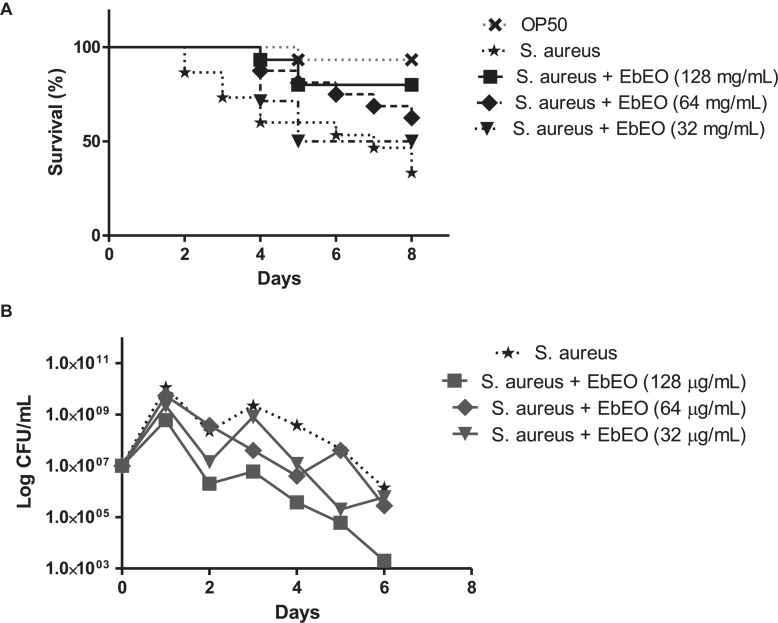
*Eugenia brejoensis* essential oil (EbEO) protects *Caenorhabditis elegans* larvae from *Staphylococcus aureus* infection. **(A)** Effects of EbEO on Survival of *C. elegans* larvae infected with *S. aureus*. **(B)** Bacterial load. * Indicates significant differences in relation to control cells (*p* < 0.05).

The infection of *C. elegans* by *S. aureus* resulted in a reduction of worm viability and 8 days post-infection (dpi) the survival ratio of this group was 30%, while the uninfected animals showed 90% survival at this day (Figure 5A). The median survival of *S. aureus*-infected worms was 7 days. This scenario was radically changed by the treatment with EbEO at 128 or 64 μg/mL. The animals treated with 128 μg/mL EbEO showed survival curve similar to control group (*p* > 0.05). In the end (8 dpi), the survival of worms treated with EbEO were around 60 and 80%, for concentrations of 64 or 128 μg/mL, respectively (Figure 5A). In addition, the *C. elegans* larvae treated with 128 μg/mL EbEO (MIC) also exhibited lower bacterial load than those incubated with 64 μg/mL EbEO (Figure 5B). It is important to highlight that the tested EbEO concentrations did not significantly reduce the viability of *C. elegans* when compared with PBS-treated group (data not shown).

### EbEO Protects *Galleria mellonella* Larvae Against the *S. aureus* Infection

**FIGURE 6:**
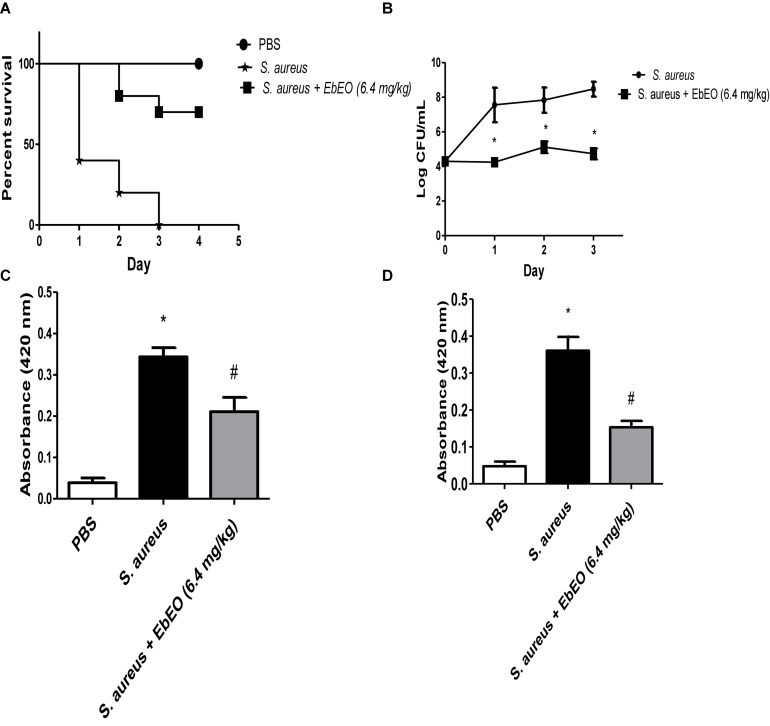
Effects of *Eugenia brejoensis* essential oil (EbEO) on *Galleria mellonella* infected with *S. aureus*. **(A)** EbEO improves the survival of *G. mellonella* infected with *S. aureus*. **(B)** EbEO reduces the load of *S. aureus* in hemolymph. **(C)** Effects of EbEO on melanin production after 1 h of *S. aureus*-infection. **(D)** Effects of EbEO on melanin production after 3 h of *S. aureus*-infection. * Indicates significant differences in relation to uninfected larvae (*p* < 0.05). # Indicates significant differences in relation to uninfected and *S. aureus-*infected larvae (*p* < 0.05).

The antibacterial activity of EbEO was further analyzed using *G. mellonella* larvae. EbEO treatment did not show any toxicity to these larvae, resulting in survival curves similar to those observed to PBS-treated animals (data not shown). The larvae infected with *S. aureus* showed 100% mortality in the third day and this group exhibited a median survival of 1 day. On the other hand, the treatment with a single dose of EbEO (6.4 mg/kg) protected 70% of the larvae at the end of the experiment (4 dpi) (Figure 6A).

Interestingly, EbEO treatment induced small effects on the bacterial load when compared to initial bacterial inoculum (time 0), suggesting a bacteriostatic action (Figure 6B). In relation to *S. aureus-*infected larvae, EbEO-treated animals exhibited significantly lower levels of bacteria in hemolymph in all days evaluated (around 3 log CFU/mL reductions for all days; *p* < 0.05).

Finally, we evaluated whether EbEO could reduce the overproduction of melanin induced by *S. aureus* infection (Figures 6C,D). We observed that the levels of melanin in the hemolymph of *S. aureus-*infected larvae were significantly increased when compared to PBS-treated group (approximately eightfolds after 1 and 3 h of infection; Figures 6C,D). The treatment with EbEO also significantly reduced the melanin content in hemolymph of *G. mellonella* larvae at both tested period (reductions of 42.19 and 55.41% after 1 and 3 h of infection, respectively; Figures 6C,D).

## Discussion

Due the high chemical diversity exhibited by EOs, they have been pointed out as interesting source of lead molecules for development of alternative antimicrobial therapies (Rubini et al., 2018; Espinoza et al., 2019; Souza Dos Santos et al., 2019). These studies have encouraged the search of new EOs from unexploited plants, and one current example is *E. brejoensis* a plant recently described in Brazil (Mazine and Souza, 2008; Giaretta and Peixoto, 2014). Herein, we reported the activity of this oil against *S. aureus*, a pathogen highlighted due its ability to harbor genes related with virulence factors and drug resistance pathways (Kos et al., 2012; Berube and Bubeck Wardenburg, 2013; Vestergaard et al., 2019).

EbEO was able to inhibit the *S. aureus* strains with MIC_50_ of 256 μg/mL. As mentioned above, EbEO is mainly composed by sesquiterpenes such as δ-cadinene, β-caryophyllene, α-muurolol, α-cadinol, and bicyclogermacrene (Da Silva et al., 2015). The anti-*S. aureus* activity of EOs containing these sesquiterpenes is well documented in the literature (Mulyaningsih et al., 2010; Salleh et al., 2015; Guerrini et al., 2016; Nishanbaev et al., 2018; Salem et al., 2018). Our results from time-kill studies demonstrated that EbEO is an antibacterial agent showing a synergetic interaction with ampicillin, chloramphenicol, and kanamycin; and additive effects with ciprofloxacin and erythromycin. The improvement of antibiotic action has been reported for several EOs and isolated sesquiterpenes (Langeveld et al., 2014; Lahmar et al., 2017; Espinoza et al., 2019; Khoury et al., 2019).

The overuse and misuse of antibiotics has resulted in different gradient of drug concentrations in humans, animals, and environment (Andersson and Hughes, 2014; Huijbers et al., 2019). In this sense, bacteria are frequently exposed to Sub-MIC of the antimicrobial agent and these conditions could result in mutagenesis and release of microbial virulence factors (Andersson and Hughes, 2014; Jo and Ahn, 2016; Larsen et al., 2016; Huijbers et al., 2019). Thus, it is important to evaluate the effects of the antimicrobial agents in pathways related to mutagenesis and virulence (Hobdey et al., 2017; Duan et al., 2018). We first showed that EbEO did not induce the expression of *recA* which is the first gene in the SOS response. The activation of SOS response occurs when bacteria need to repair damages induced in the DNA due antibiotic treatment and other adverse conditions (Simmons et al., 2008; Silva et al., 2017). The activation of SOS pathway is associated with emergence of drug resistance and acquisition of virulence factors (Andreoni et al., 2019; Meunier et al., 2019).

Our data also report that EbEO interferes with the regulation of the accessory gene regulator (*agr*)-mediated quorum sensing. This system upregulates the production of secreted virulence factors (such as α-hemolysin encoded by *hla*) and downregulates cell surface proteins (such as protein A encoded by *spa*) (Gottschalk et al., 2013; Singh and Ray, 2014). We demonstrated that sub-inhibitory concentrations of EbEO decreased the expression of *hla* and increased the expression of *spa*. Further, *S. aureus* grown in the presence of EbEO displayed reduced hemolytic activity and less ability to survival in whole blood. α-Hemolysin induces toxicity toward a broad range of cells and is associated to severe injuries in cutaneous (skin necrosis) and systemic infections (Berube and Bubeck Wardenburg, 2013; Geoghegan et al., 2018; Surewaard et al., 2018).

Other antivirulence effect of EbEO is the inhibition of STX production, a carotenoid from *S. aureus* membrane that confers protection against reactive oxygen species produced by host defense, allowing the bacteria to persist in the inflammation site (Liu and Nizet, 2009; Pannu et al., 2019). These properties make STX an attractive target for antivirulence therapy as demonstrated for some plant-derived compounds (Lee et al., 2013; Silva et al., 2017; Colasso et al., 2019). The inhibitory effects of EbEO toward STX production could be confirmed by the fact that *S. aureus* grown in the presence of this oil showed increased susceptibility to hydrogen peroxide. In addition, it may also relate to the efficacy of EbEO to rescue *C. elegans* and *G. mellonella* from *S. aureus* infection.

*Caenorhabditis elegans* is free-living terrestrial nematode considered a fast, cheap, and efficient model for *in vivo* testing of antimicrobial substances since it is susceptible to human pathogens such as *S. aureus* (Kong et al., 2014, 2016a). Our results showed that the treatment with EbEO increased the worm lifespam and decreased the bacterial load. The anti-infective efficacy of EbEO was also demonstrated using *G. mellonella* larvae. EbEO-treated animals also showed lower levels of melanin, a soluble molecule part of the humoral response of *G. mellonella* (together with lysozyme, antimicrobial peptides, and opsonins) (Zdybicka-Barabas et al., 2013; Tsai et al., 2016). The melanogenesis is part of prophenoloxidase cascade which is activated by pathogens and other foreign particles leading accumulation of nodules in order to control the microbial replication (Trevijano-Contador and Zaragoza, 2018). The melanin overproduction results in high levels of cytotoxic compounds that may induce serious damage to host tissues and cells (Zdybicka-Barabas et al., 2014). In this sense, the oil could also protect the larvae against the deleterious effects related to *S. aureus* infection.

Taken together, the results indicated the EO of *E. brejoensis* is an important source of molecules with anti-*S. aureus* action, even against MDR strains. Importantly, the oil reduced the expression of significant factors involved in *S. aureus* virulence and impaired the ability of this bacterium to survive in adverse conditions such as whole blood and under oxidative stress. The antimicrobial efficacy was confirmed using two alternative models of infections based on *C. elegans* and *G. mellonella*. These data denote the importance of prospecting new species from underexploited environmental (such as Brazilian caatinga) in order to identify new lead molecules for antimicrobial therapy. Further testing is required to know the mode of action of EbEO and details about the action of this oil against the virulence of *S. aureus* as well as individual role of the main components of the EO of *E. brejoensis* are essential for antimicrobial action.

## Data Availability Statement

The raw data supporting the conclusions of this article will be made available by the authors, without undue reservation, to any qualified researcher.

## Author Contributions

CB and LS contributed to conceptualization and investigation. AL-O, KK, MC, and MV contributed to resources. CB, LS, AL-O, and CS contributed to investigation and formal analysis. CB, LS, MV, MC, MS, and KK contributed to writing original draft. CB, MV, and LS contributed to supervision, writing-review, editing, and project administration.

## Conflict of Interest

The authors declare that the research was conducted in the absence of any commercial or financial relationships that could be construed as a potential conflict of interest.

## Footnotes

1 www.graphpad.com
